# Interactions between Neighbourhood Urban Form and Socioeconomic Status and Their Associations with Anthropometric Measurements in Canadian Adults

**DOI:** 10.1155/2017/5042614

**Published:** 2017-09-05

**Authors:** Gavin R. McCormack, Christine Friedenreich, Lindsay McLaren, Melissa Potestio, Beverly Sandalack, Ilona Csizmadi

**Affiliations:** ^1^Department of Community Health Sciences, Cumming School of Medicine, University of Calgary, Calgary, AB, Canada; ^2^Department of Oncology, Cumming School of Medicine, University of Calgary, Calgary, AB, Canada; ^3^Department of Cancer Epidemiology and Prevention Research, Cancer Control Alberta, Alberta Health Services, Edmonton, AB, Canada; ^4^Alberta Cancer Prevention Legacy Fund, Alberta Health Services, Edmonton, AB, Canada; ^5^Faculty of Environmental Design, University of Calgary, Calgary, AB, Canada

## Abstract

Neighbourhood-level socioeconomic composition and built context are correlates of weight-related behaviours. We investigated the relations between objective measures of neighbourhood design and socioeconomic status (SES) and their interaction, in relation to self-reported waist circumference (WC), waist-to-hip ratio, and body mass index (BMI) in a sample of Canadian adults (*n* = 851 from 12 Calgary neighbourhoods). WC and BMI were higher among residents of disadvantaged neighbourhoods, independent of neighbourhood design (grid, warped grid, and curvilinear street patterns) and individual-level characteristics (sex, age, education, income, dog ownership, marital status, number of dependents, motor vehicle access, smoking, sleep, mental health, physical health, and past attempts to modify bodyweight). The association between neighbourhood-level SES and WC was modified by neighbourhood design; WC was higher in disadvantaged-curvilinear neighbourhoods and lower in advantaged-grid neighbourhoods. Policies making less obesogenic neighbourhoods affordable to low socioeconomic households and that improve the supportiveness for behaviours leading to healthy weight in low socioeconomic neighbourhoods are necessary.

## 1. Introduction

The increasing global prevalence of overweight and obesity among adults and children is a major public health problem. Obesity is a risk factor for insulin resistant diabetes, hypertension, coronary heart disease, some cancers, musculoskeletal disorders, psychological disorders, gallstones, and dermatological conditions [1–4]. Because of these negative health effects, obesity imposes a significant and unsustainable economic burden on the healthcare system [5–7]. Canadian estimates suggest that in 2006 overweight and obesity accounted for 4.1% of total healthcare expenditures [8]. In the province of Alberta (Canada), the total cost attributable to obesity in 2005 was $1.27 billion [9]. Of major concern is that in 2010 18% of Canadian adults were obese; however, this is projected to increase to 21% by 2019 [10]. Alarmingly, this increasing trend in obesity will be accompanied by rising healthcare costs associated with treating and managing excess weight [11]. Obesogenic environments, which include neighbourhood built characteristics that facilitate the overconsumption of energy-dense foods and inhibit energy expenditure through reducing physical activity, have contributed to the obesity epidemic in Canada and elsewhere [12, 13].

While the maintenance of health and well-being of populations is of major importance, a pressing concern is the social and economic inequity in health. In countries such as Canada where the standards of living are relatively high, adverse health outcomes including higher rates of overweight and obesity are still found in locations with higher concentrations of low socioeconomic status individuals [14]. The mechanisms by which socioeconomic status influences obesity risk are complex [15]. Proximal determinants of weight status, including physical activity and diet, are often found to differ by socioeconomic status [16–18]. It is recognized that the environment, including physical, social, and policy settings, has major implications for obesity risk via physical activity and diet [19]. Neighbourhood design characteristics including high accessibility and affordability of calorie-dense food, reduced affordability of and reduced access to healthy food, and reduced access to physical activity opportunities may influence the relation between neighbourhood-level socioeconomic status and weight status [12].

At a population level, weight status is most often measured using self-reported body mass index (BMI). However, waist and hip circumference individually and combined (waist-to-hip ratio) reflect abdominal or visceral overweight and obesity that is not fully captured by BMI. Waist circumference and waist-to-hip ratio are independent risk factors for cardiovascular disease and type 2 diabetes [20, 21]. Moreover, waist circumference and waist-to-hip ratio may be stronger predictors of chronic disease risk and mortality, independent of BMI [22]. Waist circumference has been used previously as a measure of adiposity in high and low socioeconomic status adults [23, 24]. Studies have found associations between neighbourhood-level socioeconomic status and weight status [14, 23–25], yet few have investigated the relations between neighbourhood design and neighbourhood-level socioeconomic status and multiple measures of weight status including BMI, waist circumference, and waist-to-hip ratio, while adjusting for individual-level characteristics [24]. Thus, the aim of our study was to estimate the associations between neighbourhood design, neighbourhood-level socioeconomic status, and their interaction, in relation to waist circumference, waist-to-hip ratio, and BMI in adults.

## 2. Materials and Methods

### 2.1. Sample Design and Recruitment

The sample design and recruitment have been described elsewhere [26]. Briefly, this cross-sectional study was conducted in Calgary (Alberta, Canada) as part of the larger “Pathways to Health” project. Using stratified random sampling, 12 of the 195 established Calgary neighbourhoods built prior to 1980 were selected as recruitment sites. The 12 neighbourhood strata were defined by their block pattern (grid, warped grid, and curvilinear [27]) and socioeconomic status quartiles based on a material deprivation index described below. Using these 12 neighbourhood strata as a sampling frame allowed for the implementation of a sampling strategy that resulted in representation across the socioeconomic and urban form spectra. The City of Calgary provided an updated database containing full household address information for all dwellings located within our 12 neighbourhoods. In April 2014, a random sample of households (10,500) from each of the 12 neighbourhoods were mailed a survey package. The survey package included instructions for completing two self-administered online questionnaires: (1) a physical activity, health, and demographic questionnaire (PAHDQ) and (2) the Canadian Diet History Questionnaire II (C-DHQ II) [28]. Data from the PAHDQ only are presented herein. One adult (≥20 years of age) per household, with the next birthday, was invited to participate in the study. To encourage completion of the surveys, we offered an incentive (entry into a prize draw) and sent a warm call postcard before and two reminder postcards after sending the survey package. Of the 10,500 households sent the survey package, 407 were nondeliverable and 918 completed the online PAHDQ. An additional 105 participants requested and completed a paper copy version of the PAHDQ resulting in an estimated final response rate of 10.1% (*n* = 1023). Notably, the estimated response rate is conservative as it does not consider household access to the Internet or those ineligible to participate due to age (i.e., <20 years of age) or language barriers.

### 2.2. Variables

#### 2.2.1. Neighbourhood Design

Calgary consists of three main neighbourhood designs that are identifiable from their street pattern and which vary in regard to supportiveness for walking [27]. Our sampling framework included all three neighbourhood designs (grid, warped grid, and curvilinear). Neighbourhoods with grid street patterns include those typically built prior to 1950 and which have high street and pedestrian connectivity and permeability (e.g., predominantly four-way intersections), high mix and integration of commercial and noncommercial land uses and destinations, sidewalks on both sides of the street, treed street boulevards, and higher residential densities. Calgary neighbourhoods with a grid street pattern provide higher levels of walkability compared with the other two neighbourhood designs [29, 30]. In Calgary, neighbourhoods with warped grid patterns were typically built immediately following World War II and include streets with crescents and curves (i.e., a mix of 3- and 4-way intersections), few treed street boulevards, sidewalks located adjacent to roads, residential land uses surrounding schools or community centers, and commercial land uses located at the edge of neighbourhood. Neighbourhoods with curvilinear street patterns became the predominant Calgary suburban neighbourhood design built after 1970 to the present day. Curvilinear neighbourhoods include high-volume collector roads that link with lower volume residential roads forming a “loops and lollipops” road pattern. The curvilinear neighbourhoods have low street and pedestrian connectivity (predominantly 3-way intersection and cul-de-sacs), with sidewalks, if at all available, typically found on one side of the street only, low residential density (predominantly single-family dwellings), and a lack of integration of residential and commercial land uses with clusters of commercial land uses surrounded by parking lots (i.e., strip malls) located on the edge of the neighbourhood located on a high-volume collector road. However, curvilinear neighbourhoods often include large areas of green space and park areas. Compared with grid and warped grid street pattern neighbourhoods, curvilinear neighbourhoods in Calgary have been found to offer the least built support for utilitarian walking [29]. This study focusses on neighbourhood design instead of specific built characteristics because Calgary neighbourhoods can be easily identified by their street patterns which may facilitate targeted interventions.

#### 2.2.2. Neighbourhood-Level Socioeconomic Status

Informed by previous findings [31], we undertook principal component analysis with seven variables from the Statistics Canada 2006 national census that reflected neighbourhood social and material deprivation (proportion of 25–64-year-olds whose highest education is below a high school diploma; proportion of single-parent families; proportion of rented private dwellings; proportion of the divorced, separated, or widowed among those ≥15 years of age; proportion of the unemployed among those ≥25 years of age; median gross household income; and average value of dwellings). Prior to undertaking the principal component analysis, the census dissemination area level variables were aggregated to the neighbourhood administrative boundary level and converted to *z*-scores. From the principal component analysis results, a single socioeconomic deprivation index was identified (explained variance = 50.1%; factor loadings for the 7 variables = 0.51 to 0.83). The socioeconomic deprivation index was divided into quartiles and neighbourhoods labelled as “advantaged,” “somewhat advantaged,” “somewhat disadvantaged,” and “disadvantaged.” These results have been presented elsewhere [26].

#### 2.2.3. Weight Status Outcomes

Participants reported their height and weight and measured their waist and hip circumferences. Height is often overestimated while weight underestimated among certain populations, particularly among obese individuals [32, 33]; thus we captured multiple anthropometric measures to improve the validity of our findings. BMI was estimated from self-reported height and weight (weight in kilograms/height in meters^2^). Participants were provided with written instructions and diagrams explaining how to undertake the waist and hip circumference measurements. Using a clinical grade measuring tape (Medline, Model NON171333), participants measured and recorded their waist and hip circumferences. Participants measured and reported each measurement twice (or a third time was required if the difference between the first two measurements was >0.50 centimeters), from which we estimated the average waist and hip circumferences. Participants were instructed to measure their waist circumference with the tape placed 2 centimeters (cm) above their navel and to measure their hip circumference at the largest location between their hips and thighs. The average waist and hip circumferences were used to estimate waist-to-hip ratio. Acceptable levels of concurrent validity have been found between self-measured and technician-measured waist and hip circumference [34–36]. Three anthropometric measures (BMI, waist circumference, and waist-to-hip ratio) were examined as continuous outcome variables.

#### 2.2.4. Sociodemographic and Health Covariates

The PAHDQ included sociodemographic items that captured participant's sex, age, ethnicity (white or other), highest education attained (high school or less, college, or university), gross annual household income (<$60,000, $60,000 to 119,000, ≥$120,000, or do not know/refused to answer), marital status (married/common law or other), number of children at home <18 years of age (at least one or none), dog ownership in the past 12 months (owner or nonowner), and motor vehicle access (always/sometimes or never/do not drive). In addition, the questionnaire captured health characteristics including smoking of cigarettes or tobacco in the past 12 months (daily/occasionally or not at all), typical amount of time per day spent sleeping, self-reported mental health and self-reported physical health (measured on a 5-point scale: poor, fair, good, very good, and excellent), and whether or not the participant had attempted to modify their weight in the past 12 months using any approach (diet, physical activity, supplements, and surgery).

### 2.3. Statistical Analysis

Descriptive analysis including the estimation of means, standard deviations, and frequencies was undertaken for all neighbourhood-level, sociodemographic, and anthropometric variables for participants with complete and missing data. The sample characteristics were descriptively compared with similar characteristics from the 2014 Calgary Civic Census for the 12 study neighbourhoods including age, sex, visible minority, education, income, marital status, and number of children at home. Pearson's correlations were estimated between the anthropometric outcomes (BMI, waist circumference, and waist-to-hip ratio).

Multivariable generalized linear regression (with normal distribution, identity link function, and Huber-White standard errors) was used to estimate unstandardized beta (*β*) coefficients and 95 percent confidence intervals (95% CI) for the associations between each of the anthropometric outcomes (BMI, waist circumference, and waist-to-hip ratio) and neighbourhood design and neighbourhood-level socioeconomic status. These models were further adjusted by the addition of all sociodemographic and health covariates. Main effects with *p* values less than 0.05 were considered statistically significant.

Interaction terms capturing the interrelationship between neighbourhood design and neighbourhood-level socioeconomic status were also estimated for each of the fully adjusted models. For statistically significant interaction effects (*p* < 0.10), we estimated the marginal means for each combination of neighbourhood design (grid, warped grid, and curvilinear) and neighbourhood-level socioeconomic status (advantaged, somewhat advantaged, somewhat disadvantaged, and disadvantaged), undertook pairwise comparisons (Fisher's Least Significant Difference), and presented these results in graph form using Microsoft Excel.

## 3. Results

### 3.1. Sample Characteristics

Complete data were available for *n* = 851 participants (*n* = 749 online and *n* = 102 hardcopy versions of the questionnaire). Compared with participants who provided complete data, those with incomplete data were significantly (*p* < 0.05) older, reported worse self-reported physical health, were less likely to be a dog owner, to have access to a motor vehicle, to be married/common law, to have children <18 years of age, or to have a postsecondary education, and had larger waist circumference. Compared with the census population data averaged across the 12 study neighbourhoods, our sample was older and had higher income and included higher proportions of women, whites, those completing postsecondary education, those married or common law, and those without children <18 years of age at home (Table 1).

Our sample consisted mostly of women, those with a university level education, those residing in a household with an income ≥$120,000/year, married or common law, parents of children at home, dog owners, those with access to a motor vehicle, and nonsmokers (Table 1). Further, approximately one-half of participants reported attempting to change their weight in the past 12 months. Mean (±standard deviation) BMI was 24.9 ± 4.9 kg/m^2^, waist circumference was 86.5 ± 13.7 cm, and waist-to-hip ratio was 0.88 ± 0.13 (Table 1). Despite no differences in BMI or waist circumference between online and hardcopy participants, the difference in waist-to-hip ratio reached statistical significance (88.2 ± 12.2 versus 90.8 ± 15.8, *p* < 0.05). The correlations between the anthropometric outcomes were positive and statistically significant (*p* < 0.05; BMI by waist circumference: *r* = 0.63; BMI by waist-to-hip ratio: *r* = 0.32; waist circumference by waist-to-hip ratio: *r* = 0.61).

### 3.2. Correlates of Waist Circumference

Adjusting for the neighbourhood-level socioeconomic status only, participants residing in curvilinear neighbourhoods, on average, had significantly (*p* < 0.05) larger waist circumference (*β* = 4.09; 95% CI 1.68, 6.50 cm) than participants from grid neighbourhoods (Table 2). Adjusting for neighbourhood design only, compared with participants from the most disadvantaged neighbourhood, those residing in the advantaged neighbourhoods, on average, had smaller waist circumference (advantaged: *β* = −3.80; 95% CI −6.98, −0.63 cm).

After adjustment for all covariates, neighbourhood design was no longer significantly associated with waist circumference; however, there remained significant (*p* < 0.05) differences in waist circumference with advantaged (*β* = −7.73; 95% CI −7.38, −2.08 cm) and somewhat advantaged (*β* = −4.17; 95% CI −6.95, −1.39 cm) neighbourhoods having significantly lower waist circumference compared with disadvantaged neighbourhoods (Table 2).

**Figure 1:**
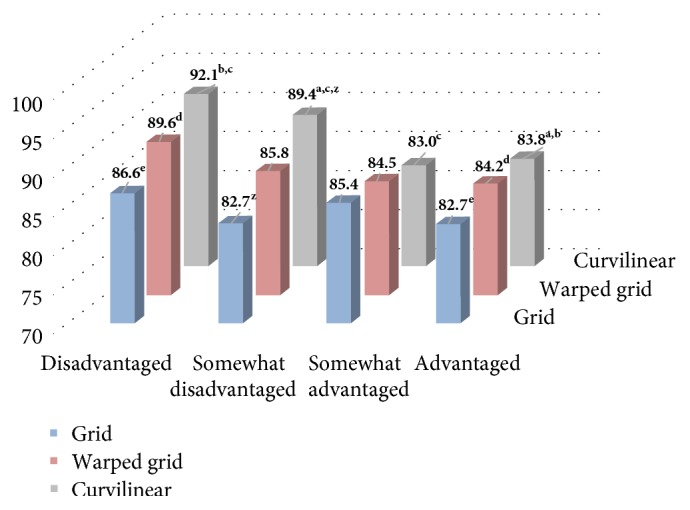
Fully adjusted estimate marginal means of waist circumference for combinations of neighbourhood urban form (grid, warped grid, and curvilinear) and socioeconomic status (disadvantaged, somewhat disadvantaged, somewhat advantaged, and advantaged) based on significant interaction term (*p* < 0.10). Columns with the same superscript are significantly different based on Fisher's Least Significance test (*p* < 0.05).

We found a significant interaction between neighbourhood design and neighbourhood-level socioeconomic status (*p* = 0.08). For grid, warped grid, and curvilinear neighbourhood designs, compared with those residing in advantaged neighbourhoods, those residing in disadvantaged neighbourhoods had significantly higher waist circumference (*p* < 0.05) with the highest found for disadvantaged-curvilinear neighbourhoods (92.1 cm) and lowest for advantaged-grid neighbourhoods (82.7 cm) (Figure 1). Curvilinear neighbourhoods had higher average waist circumference compared with grid neighbourhoods for those that were somewhat disadvantaged (89.4 versus 82.7 cm, *p* < 0.05).

### 3.3. Correlates of Waist-to-Hip Ratio

Neither neighbourhood design nor neighbourhood-level socioeconomic status was associated with waist-to-hip ratio (Table 2). The interaction between neighbourhood design and neighbourhood-level socioeconomic status for waist-to-hip ratio was not significant (*p* = 0.11).

### 3.4. Correlates of Body Mass Index

Adjusting for the neighbourhood-level socioeconomic status only, compared with participants residing in grid neighbourhoods, those residing in curvilinear and warped grid neighbourhoods, on average, had higher (*p* < 0.05) BMI (*β* = 1.39; 95% CI 0.52, 2.26 and *β* = 0.89; 95% CI 0.04. 1.75, resp.) (Table 2). Adjusting for neighbourhood design only, compared with participants from the most disadvantaged neighbourhood, on average, those residing in somewhat advantaged (*β* = −1.43; 95% CI −2.68, −0.18), and advantaged (*β* = −2.02; 95% CI −3.16, −0.89) neighbourhoods had significantly (*p* < 0.05) lower BMI. After adjustment for all covariates, neighbourhood design was no longer significantly associated with BMI; however, there remained significant (*p* < 0.05) differences in BMI with advantaged (*β* = −2.27; 95% CI −3.40, −1.13) and somewhat advantaged (*β* = −1.80; 95% CI −3.01, −0.59) neighbourhoods having significantly lower BMI compared with disadvantaged neighbourhoods (Table 2). The neighbourhood design by neighbourhood-level socioeconomic status interaction for BMI was not significant (*p* = 0.26).

## 4. Discussion

Like other studies [14, 24, 25], we found that higher neighbourhood-level socioeconomic status, independent of individual characteristics and neighbourhood design, was associated with a healthier weight status, including smaller self-reported waist circumference and lower self-reported BMI. Contributing to the equivocal evidence [37–39], we also found that neighbourhood design was not independently associated with weight status. However, a novel study finding included the significant interaction between neighbourhood-level socioeconomic status and neighbourhood design and their joint association with self-reported waist circumference.

Given the mixed evidence regarding the built environment's contribution to weight status [37–39], the null association found between neighbourhood design and weight status in our study was not surprising. We found that individuals residing in curvilinear neighbourhoods had larger waist circumferences and higher BMI levels but the strength of associations was attenuated after adjusting for covariates. This finding might suggest that these individual-level factors are associated with residential self-selection. Weight status is associated with physical activity, and associations between physical activity and neighbourhood built characteristics are often found to attenuate after adjustment for measures of residential self-selection [40, 41]. Notably, Martin et al. [42] in their recent review found little evidence for overestimated associations between the built environment and weight status in analysis that did not statistically adjust for residential self-selection. Another reason for the null association could be due to our general measure of neighbourhood design that was classified by street pattern (grid, warped grid, and curvilinear). While neighbourhoods defined by their street pattern may be easily identifiable for intervention purposes, between neighbourhoods with the same street pattern there are likely differences in other built characteristics that might influence weight status or weight-related behaviours (e.g., diet and physical activity) [38]. Nevertheless, Sugiyama et al. [38]. found that, among the studies that reported an association between the built environment and weight status, composite built environment variables (e.g., “walkability”) and the availability of utilitarian destinations such as shops and services were the most consistent correlates. Grid neighbourhoods in Calgary have high pedestrian connectivity and include a mix of shops and services as well as other features typically found in “walkable” neighbourhoods (e.g., high population densities, sidewalks). We will explore the influence of neighbourhood built characteristics other than street pattern on weight status in future research.

Associations between neighbourhood-level socioeconomic status and waist circumference and BMI did not attenuate after adjustment for covariates. Other studies controlling for neighbourhood environment and individual-level characteristics have found lower neighbourhood-level socioeconomic status to be associated with weight gain [43, 44]. We found that, on average, waist circumference was approximately 4 cm smaller and BMI 2 kg/m^2^ units lower among residents of advantaged versus disadvantaged neighbourhoods. This difference in weight status could have population health implications given that in some adult populations differences of approximately 1 cm in waist circumference and 1 kg/m^2^ in BMI have been associated with an increased risk of type II diabetes [45], cardiovascular disease [20], and mortality [46]. Thus, the difference in waist circumference and BMI between advantaged and disadvantaged neighbourhoods of the magnitude observed in our study could lead to health disparities.

Similar to other Canadian studies [47], we found associations between individual-level correlates and weight status independent of neighbourhood-level characteristics, including sex, age, dependents, smoking status, and self-reported health. Our findings suggest that, regardless of the neighbourhood design or socioeconomic characteristics, interventions for promoting healthy weight should specifically target higher obesity-risk populations including men, older adults, those without children at home, and those with poor physical health. Notably, we found that better self-reported mental health was associated with larger waist circumference and BMI despite previous evidence suggesting that poorer mental health including anxiety [48] and depression [49] are positively associated with weight status. While intuitive, we also found that participants who attempted to modify their weight in the past year had higher waist circumference, waist-to-hip ratio, and BMI.

Furthermore, our findings suggest that neighbourhood design may exacerbate the impact neighbourhood-level socioeconomic status has on waist circumference. Within the most disadvantaged neighbourhoods, waist circumference was lower for grid versus curvilinear neighbourhoods. Neighbourhood design may be even more important for health for those residing in low socioeconomic status neighbourhoods. Others have found that food stores, exercise facilities, and safety might have a stronger impact on obesity among those residing in low socioeconomic neighbourhoods [50]. Characteristics such as sidewalk availability and condition (i.e., obstructions and unevenness) and physical disorder (i.e., trash, graffiti, and neglected properties) are sometimes worse in low versus high socioeconomic status neighbourhoods [51]. Furthermore, the presence of recreational facilities such as fitness and dance facilities, sports and recreation clubs, and golf courses is often less likely to be located in socioeconomically disadvantaged neighbourhoods [52]. In addition, residents of low socioeconomic status neighbourhoods typically have increased accessibility to shops offering unhealthy foods [53] and reduced accessibility to supermarkets and shops offering fresh fruit and vegetables [54]. Steps to make disadvantaged neighbourhoods more “grid-like” or less obesogenic could promote healthy weight and or reduce overweight and obesity in this vulnerable population. Some evidence suggests that in North America more walkable (or “less obesogenic”) neighbourhoods have higher property values [55, 56] potentially making these healthier neighbourhoods less affordable to live in for low socioeconomic status households. Improving neighbourhood design to make it less obesogenic (i.e., improved availability and access to healthy food choices and physical activity opportunities) might also increase residential property values and rents, in turn displacing socioeconomic status households who can no longer afford to reside in the gentrified neighbourhood [57, 58]. Policymakers, developers, urban planners, and financial institutions need to consider exploring and implementing potential strategies (e.g., location-based mortgages, subsidized housing or rent, increased density of smaller housing units, and increased availability of publicly funded housing) that increase the affordability of owning or renting residential property in health supportive neighbourhoods for low socioeconomic households [57, 58].

Although our findings are encouraging, we acknowledge that the cross-sectional design limits the causal inferences that can be drawn. It is possible that people with preferences for certain types of weight-related behaviours (e.g., healthy diets and physical activity) may choose residential neighbourhoods that match their preferences (residential self-selection) [40], although this may be less of an issue for weight status outcomes [41]. Further, the low response rate, the differences between participants and nonparticipants, the differences between participants who provided complete and incomplete data, our sample design, which included 12 established Calgary neighbourhoods, and our data collection approach (primarily online data collection) limit the generalizability of our findings. While biases in self-assessment of waist circumference and height and weight exist [23], our inclusion of three measures of weight status (waist circumference, waist-to-hip ratio, and BMI) and our objective measures of neighbourhood design and socioeconomic status is a study strength. Our study focused on a “macro” level indicator of the built environment only, specifically neighbourhood design or street pattern. However, we acknowledge that “micro” level built characteristics, often captured via in-person or virtual audits (e.g., sidewalk condition, signage, street furniture, aesthetics, safety features, and the presence of local food and physical activity related destinations), found within local streets, could influence weight influencing behaviours, such as physical activity and diet. Future studies should consider investigating the combined influence of macro and micro level neighbourhood built characteristics on adiposity, physical activity, and diet.

## 5. Conclusions

Our findings suggest that, in addition to building neighbourhoods that support physical activity and healthy diet, specific weight reduction or obesity prevention interventions may need to be implemented in existing curvilinear neighbourhoods with higher concentrations of socioeconomically disadvantaged households.

## Figures and Tables

**Table 1:** Sample profile of sociodemographic, health, weight status, and neighbourhood environment variables for participants with complete and incomplete data.

Characteristics	Category	Sample with complete data for all variables used in analysis		Census statistics for study neighbourhoods (*n* = 12)^*∗*^
		*n*	Estimate	Estimate
Age in years [mean ± SD]		851	52.8 ± 14.3	39 years (median age)
Sex [%]	Women	851	62.4	50.3% (men ≥ 20 years of age)
	Men		37.6	49.7% (women ≥ 20 years of age)
Ethnicity [%]	White	851	88.4	
	Nonwhite		11.6	22.4% visible minority
Highest education achieved [%]	High school or less	851	7.6	36% high school or less
	College		19.2	
	University		73.2	63% postsecondary
Gross annual household income [%]	<$60,000	851	10.6	$85,478 (median income)
	60–119,000		30.1	
	≥120,000		44.1	
	Do not know/refused		15.3	
Marital status [%]	Married/common law	851	77.3	55.6% married
	Other arrangement		22.7	
Number of children < 18 years of age [%]	At least one child	851	31.0	47.5% with child at home
	No children		69.0	
Dog ownership in the past year [%]	Owner	851	32.7	
	Nonowner		67.3	
Motor vehicle access [%]	Always/sometimes	851	86.6	
	Never/do not drive		13.4	
Smoking in the past year [%]	Daily/occasionally	851	5.3	
	Not at all		94.7	
Sleeping hours/day [mean ± SD]		851	7.3 ± 1.0	
Self-reported mental health [mean ± SD]		851	3.9 ± 0.9	
Self-reported physical health [mean ± SD]		851	3.8 ± 0.9	
Weight modification in the past year [%]	Attempted	851	51.0	
	Not attempted		49.0	
Waist circumference [mean ± SD]		851	86.5 ± 13.7	
Waist-to-hip ratio [mean ± SD]		851	0.88 ± 0.13	
Body mass index [mean ± SD]		851	24.9 ± 4.9	
Neighbourhood design [%]	Curvilinear	851	33.3	
	Warped grid		38.2	
	Grid		28.6	
Neighbourhood SES quartiles [%]	Disadvantaged	851	13.9	
	Somewhat disadvantaged		19.7	
	Somewhat advantaged		27.4	
	Advantaged		39.0	

^*∗*^Data from the 2014 Calgary Civic Census and estimates based on unweighted average of aggregate data across the 12 study neighbourhoods; SD: standard deviation.

**Table 2:** Multivariable linear regression unstandardized beta (*β*) coefficients and 95 percent confidence intervals (95 CI) for the associations between weight status and sociodemographic, health, and neighbourhood variables (*n* = 851).

Characteristics	Category	Waist circumference Unstandardized *β* (95 CI)		Waist-to-hip ratio^*∗∗*^ Unstandardized *β* (95 CI)		Body mass index Unstandardized *β* (95 CI)	
		Model 1	Model 2	Model 1	Model 2	Model 1	Model 2
*Intercept*		*86.76 (83.94, 89.58)*	*90.02 (81.36, 98.67)*	*88.89 (87.24, 90.51)*	*83.08 (76.82, 89.34)*	*25.84 (24.38, 26.59)*	*27.04 (23.57, 30.51)*
Neighbourhood design	Curvilinear	4.09(1.68,6.50)^*∗*^	0.79 (−1.20, 2.79)	−2.31 (−4.89, 0.27)	0.33 (−1.87, 2.54)	1.39(0.52,2.26)^*∗*^	0.55 (−0.30, 1.40)
	Warped grid	2.22 (−0.15, 4.58)	0.56 (−1.36, 2.47)	−1.54 (−3.46, 0.35)	0.51 (−1.22, 2.25)	0.89(0.04,1.75)^*∗*^	0.41 (−0.41, 1.23)
	Grid	0	0	0		0	0
Neighbourhood SES quartiles	Advantaged	−3.80(−6.98, −0.63)^*∗*^	−4.73(−7.38, −2.08)^*∗*^	1.56 (−1.36, 4.49)	−0.67 (−3.10, 1.76)	−2.02(−3.16, −0.89)^*∗*^	2.27(−3.40, −1.13)^*∗*^
	Somewhat advantaged	−2.86 (−6.22, 0.50)	−4.17(−6.95, −1.39)^*∗*^	1.53 (−0.62, 3.69)	0.06 (−2.95, 3.08)	−1.43(−2.68, −0.18)^*∗*^	−1.80(−3.01, −0.59)^*∗*^
	Somewhat disadvantaged	−0.80 (−4.56, 2.96)	−1.86 (−4.84, 1.11)	1.27 (−0.96, 3.50)	0.46 (−2.06, 2.97)	−0.89(−2.20,0.42)^*∗*^	−1.12 (−2.33, 0.09)
	Disadvantaged	0	0	0	0	0	0
Age in years			0.22(0.16,0.28)^*∗*^		0.13(0.06,0.21)^*∗*^		0.04(0.02,0.07)^*∗*^
Sex	Men		11.95(10.45,13.46)^*∗*^		13.95(12.65,15.25)^*∗*^		1.66(1.03,2.29)^*∗*^
	Women		0		0		0
Ethnicity	Nonwhite		−2.23 (−4.50, 0.04)		2.20 (−0.85, 5.25)		−0.23 (−1.24, 0.78)
	White		0		0		0
Highest education achieved	High school or less		−0.52 (−3.38, 2.35)		4.92(0.70,9.14)^*∗*^		−0.98 (−2.04, 0.09)
	College		1.21 (−0.93, 3.34)		0.79, (−0.72, 2.30)		−0.00 (−0.91, 0.91)
	University		0		0		0
Gross annual household income	Do not know/refused		−1.25 (−3.11, 0.61)		−0.46 (−2.03, 1.12)		0.20 (−0.86, 1.26)
	<$60,000		0.93 (−1.87, 3.74)		−0.31 (−3.01, 2.40)		0.49 (−0.89, 1.88)
	60−119,000		−0.09 (−2.02, 1.84)		1.00 (−0.48, 2.49)		−0.38 (−1.09, 0.34)
	≥120,000		0		0		0
Marital status	Married/common law		0.26 (−1.60, 2.12)		−0.36 (−1.99, 1.26)		0.07 (−0.70, 0.85)
	Other arrangement		0		0		0
Number of children < 18 years of age	No children		1.46 (−0.29, 3.20)		0.17 (−1.40, 1.74)		0.85(0.14,1.56)^*∗*^
	At least one child		0		0		0
Dog ownership in the past year	Nonowner		−0.02 (−1.48, 1.45)		−0.27 (−1.77, 1.23)		−0.05 (−0.68, 0.59)
	Owner		0		0		0
Motor vehicle access	Never or do not drive		−2.81 (−5.74, 0.12)		1.58 (−1.24, 4.41)		−0.39 (−1.41, 0.64)
	Always/sometimes		0		0		0
Smoking in the past year	Daily/occasionally		−1.54 (−5.75, 2.66)		2.82 (−3.16, 8.79)		−0.96(−1.88, −0.05)^*∗*^
	Not at all		0		0		0
Sleeping hours/day			−0.88 (−1.93, 0.17)		−0.23 (−0.97, 0.51)		−0.12 (−0.51, 0.26)
Self-reported mental health			1.33(0.30,2.36)^*∗*^		−0.59 (−2.21, 0.94)		0.48(0.01,0.95)^*∗*^
Self-reported physical health			−3.76(−4.76, −2.76)^*∗*^		0.82 (−1.76, 0.12)		−1.21(−1.64, −0.78)^*∗*^
Weight modification in the past year	Not attempted		−4.36(−5.81, −2.90)^*∗*^		−1.70(−3.05, −0.34)^*∗*^		−1.49(−2.11, −0.87)^*∗*^
	Attempted		0		0		0

Model 1 adjusted for neighbourhood street pattern and neighbourhood level socioeconomic status only; Model 2 adjusted for neighbourhood street pattern and neighbourhood level socioeconomic status plus all sociodemographic and health variables; ^*∗*^*p* < 0.05; ^*∗∗*^rescaled to 100 to aid in interpretation of estimates.

## Additional Points

*Highlights*. (i) Participants in advantaged neighbourhoods had smaller waist circumference and BMI. (ii) Neighbourhood design and socioeconomic status jointly affect waist circumference. (iii) Neighbourhood and individual characteristics independently influence weight status.

## References

[1] Simon G. E., Von Korff M., Saunders K. (2006). Association between obesity and psychiatric disorders in the US adult population. *Archives of General Psychiatry*.

[2] Yosipovitch G., DeVore A., Dawn A. (2007). Obesity and the skin: skin physiology and skin manifestations of obesity. *Journal of the American Academy of Dermatology*.

[3] Pi-Sunyer F. X., Fairburn C. G., Brownell K. D. (2002). Medical complications of obesity in adults. *Eating Disorders And Obesity*.

[4] McLaren L., Beck C. A., Patten S. B., Fick G. H., Adair C. E. (2008). The relationship between body mass index and mental health. *Social Psychiatry and Psychiatric Epidemiology*.

[5] Katzmarzyk P. T., Janssen I. (2004). The economic costs associated with physical inactivity and obesity in Canada: an update. *Canadian Journal of Applied Physiology*.

[6] Finkelstein E. A., Fiebelkorn I. C., Wang G. National medical spending attributable to overweight and obesity: how much, and who's paying?. *Health Aff (Millwood)*.

[7] Kouris-Blazos A., Wahlqvist M. L. (2007). Health economics of weight management: evidence and cost. *Asia Pacific Journal of Clinical Nutrition*.

[8] Anis A. H., Zhang W., Bansback N., Guh D. P., Amarsi Z., Birmingham C. L. (2010). Obesity and overweight in Canada: an updated cost-of-illness study. *Obesity Reviews*.

[9] Colman R., Hayward K. (2010). *The Cost od Obesity in Alberta Summary Report*.

[10] Twells L. K., Gregory D. M., Reddigan J., Midodzi W. K. (2014). Current and predicted prevalence of obesity in Canada: a trend analysis. *CMAJ Open*.

[11] Flegal K. M., Carroll M. D., Ogden C. L., Curtin L. R. (2010). Prevalence and trends in obesity among US adults, 1999–2008. *Journal of the American Medical Association*.

[12] French S. A., Story M., Jeffery R. W. (2001). Environmental influences on eating and physical activity. *Annual Review of Public Health*.

[13] Brownell K. D. (2003). *European Eating Disorders Review*.

[14] Ross N. A., Tremblay S., Khan S., Crouse D., Tremblay M., Berthelot J.-M. (2007). Body mass index in urban Canada: Neighborhood and metropolitan area effects. *American Journal of Public Health*.

[15] McLaren L., Godley J., MacNairn I. (2009). Social class, gender, and time use: implications for the social determinants of body weight?. *Health Reports*.

[16] Ball K., Crawford D. (2005). Socioeconomic status and weight change in adults: a review. *Social Science and Medicine*.

[17] Sobal J., Stunkard A. J. (1989). Socioeconomic status and obesity: a review of the literature. *Psychological Bulletin*.

[18] McLaren L. (2007). Socioeconomic status and obesity. *Epidemiologic Reviews*.

[19] Hill J. O., Peters J. C. (1998). Environmental contributions to the obesity epidemic. *Science*.

[20] de Koning L., Merchant A. T., Pogue J., Anand S. S. (2007). Waist circumference and waist-to-hip ratio as predictors of cardiovascular events: meta-regression analysis of prospective studies. *European Heart Journal*.

[21] Snijder M. B., Dekker J. M., Visser M. (2003). Associations of hip and thigh circumferences independent of waist circumference with the incidence of type 2 diabetes: the Hoorn study. *The American Journal of Clinical Nutrition*.

[22] Leitzmann M. F., Moore S. C., Koster A. (2011). Waist circumference as compared with body-mass index in predicting mortality from specific causes. *PLoS ONE*.

[23] Dekkers J. C., Van Wier M. F., Hendriksen I. J. M., Twisk J. W. R., Van Mechelen W. (2008). Accuracy of self-reported body weight, height and waist circumference in a Dutch overweight working population. *BMC Medical Research Methodology*.

[24] Ellaway A., Anderson A., Macintyre S. (1997). Does area of residence affect body size and shape?. *International Journal of Obesity*.

[25] Van Lenthe F. J., Mackenbach J. P. (2002). Neighbourhood deprivation and overweight: The GLOBE study. *International Journal of Obesity*.

[26] McInerney M., Csizmadi I., Friedenreich C. M. (2016). Associations between the neighbourhood food environment, neighbourhood socioeconomic status, and diet quality: An observational study. *BMC Public Health*.

[27] Sandalack B., Nicolai A. (2006). *The Calgary Project: Urban Form/Urban Life*.

[28] Csizmadi I., Boucher B. A., Lo Siou G. (2016). Using national dietary intake data to evaluate and adapt the US Diet History Questionnaire: The stepwise tailoring of an FFQ for Canadian use. *Public Health Nutrition*.

[29] McCormack G. R., Friedenreich C., Sandalack B. A., Giles-Corti B., Doyle-Baker P. K., Shiell A. (2012). The relationship between cluster-analysis derived walkability and local recreational and transportation walking among Canadian adults. *Health and Place*.

[30] Sandalack B. A., Alaniz Uribe F. G., Eshghzadeh Zanjani A., Shiell A., McCormack G. R., Doyle-Baker P. K. (2013). Neighbourhood type and walkshed size. *Journal of Urbanism*.

[31] Pampalon R., Raymond G. (2000). A deprivation index for health and welfare planning in Quebec. *Chronic Diseases in Canada*.

[32] Gorber S. C., Tremblay M., Moher D., Gorber B. (2007). A comparison of direct vs. self-report measures for assessing height, weight and body mass index: a systematic review. *Obesity Reviews*.

[33] Akhtar-Danech N., Dehghan M., Merchant A., Rainey J. (2008). Valdity pf self-reported height and weight for measuring prevalence of obesity.Open Medicine. *Rainey J: Valdity pf self-reported height and weight for measuring prevalence of obesity. Open Medicine*.

[34] Spencer E. A., Roddam A. W., Key T. J. (2004). Accuracy of self-reported waist and hip measurements in 4492 EPIC-Oxford participants. *Public Health Nutrition*.

[35] Kushi L. H., Kaye S. A., Folsom A. R., Soler J. T., Prineas R. J. (1988). Accuracy and reliability of self-measurement of body girths. *American Journal of Epidemiology*.

[36] van den Donk M., Bobbink I. W. G., Gorter K. J., Salomé P. L., Rutten G. E. H. M. (2009). Identifying people with metabolic syndrome in primary care by screening with a mailed tape measure. A survey of 14,000 people in the Netherlands. *Preventive Medicine*.

[37] Durand C. P., Andalib M., Dunton G. F., Wolch J., Pentz M. A. (2011). A systematic review of built environment factors related to physical activity and obesity risk: Implications for smart growth urban planning. *Obesity Reviews*.

[38] Sugiyama T., Koohsari M. J., Mavoa S., Owen N. (2014). Activity-Friendly Built Environment Attributes and Adult Adiposity. *Current Obesity Reports*.

[39] Feng J., Glass T. A., Curriero F. C., Stewart W. F., Schwartz B. S. (2010). The built environment and obesity: A systematic review of the epidemiologic evidence. *Health and Place*.

[40] Boone-Heinonen J., Gordon-Larsen P., Guilkey D. K., Jacobs D. R., Popkin B. M. (2011). Environment and physical activity dynamics: The role of residential self-selection. *Psychology of Sport and Exercise*.

[41] McCormack G. R., Shiell A. (2011). In search of causality: a systematic review of the relationship between the built environment and physical activity among adults. *International Journal of Behavioral Nutrition and Physical Activity*.

[42] Martin A., Ogilvie D., Suhrcke M. (2014). Evaluating causal relationships between urban built environment characteristics and obesity: A methodological review of observational studies. *International Journal of Behavioral Nutrition and Physical Activity*.

[43] Powell-Wiley T. M., Cooper-McCann R., Ayers C. (2015). Change in Neighborhood Socioeconomic Status and Weight Gain: Dallas Heart Study. *American Journal of Preventive Medicine*.

[44] Smith K. R., Brown B. B., Yamada I., Kowaleski-Jones L., Zick C. D., Fan J. X. (2008). Walkability and body mass index. density, design, and new diversity measures. *American Journal of Preventive Medicine*.

[45] Feller S., Boeing H., Pischon T. (2010). Body mass index, waist circumference, and the risk of type 2 diabetes mellitus: Implications for routine clinical practice. *Deutsches Arzteblatt*.

[46] Jacobs E. J., Newton C. C., Wang Y. (2010). Waist circumference and all-cause mortality in a large US cohort. *Archives of Internal Medicine*.

[47] Pouliou T., Elliott S. J. (2010). Individual and socio-environmental determinants of overweight and obesity in Urban Canada. *Health and Place*.

[48] Gariepy G., Nitka D., Schmitz N. (2010). The association between obesity and anxiety disorders in the population: a systematic review and meta-analysis. *International Journal of Obesity*.

[49] Luppino F. S., de Wit L. M., Bouvy P. F. (2010). Overweight, obesity, and depression: a systematic review and meta-analysis of longitudinal studies. *Archives of General Psychiatry*.

[50] Lovasi G. S., Hutson M. A., Guerra M., Neckerman K. M. (2009). Built environments and obesity in disadvantaged populations. *Epidemiologic Reviews*.

[51] Kelly C. M., Schootman M., Baker E. A., Barnidge E. K., Lemes A. (2007). Evidence-Based Public Health Policy and Practice: the association of sidewalk walkability and physical disorder with area-level race and poverty. *Journal of Epidemiology and Community Health*.

[52] Powell L. M., Slater S., Chaloupka F. J., Harper D. (2006). Availability of physical activity-related facilities and neighborhood demographic and socioeconomic characteristics: A national study. *American Journal of Public Health*.

[53] Block J. P., Scribner R. A., Desalvo K. B. (2004). Fast food, race/ethnicity, and income: A geographic analysis. *American Journal of Preventive Medicine*.

[54] Powell L. M., Slater S., Mirtcheva D., Bao Y., Chaloupka F. J. (2007). Food store availability and neighborhood characteristics in the United States. *Preventive Medicine*.

[55] Gilderbloom J. I., Riggs W. W., Meares W. L. (2015). Does walkability matter? An examination of walkability's impact on housing values, foreclosures and crime. *Cities*.

[56] Li W., Joh K., Lee C., Kim J.-H., Park H., Woo A. (2015). Assessing Benefits of Neighborhood Walkability to Single-Family Property Values: A Spatial Hedonic Study in Austin, Texas. *Journal of Planning Education and Research*.

[57] Talen E. (2013). Prospects for walkable, mixed-income neighborhoods: Insights from U.S. developers. *Journal of Housing and the Built Environment*.

[58] Talen E., Koschinsky J. (2013). The Walkable Neighborhood: A Literature Review. *International Journal of Sustainable Land Use and Urban Planning*.

